# Dry Reforming of Methane over 5%Ni/Ce_1-x_Ti_x_O_2_ Catalysts Obtained via Synthesis in Supercritical Isopropanol

**DOI:** 10.3390/ijms24119680

**Published:** 2023-06-02

**Authors:** Ekaterina Smal, Yulia Bespalko, Marina Arapova, Valeria Fedorova, Konstantin Valeev, Nikita Eremeev, Ekaterina Sadovskaya, Tamara Krieger, Tatiana Glazneva, Vladislav Sadykov, Mikhail Simonov

**Affiliations:** Department of Heterogeneous Catalysis, Boreskov Institute of Catalysis, Pr. Akademika Lavrentieva, 5, 630090 Novosibirsk, Russia; bespalko@catalysis.ru (Y.B.); arapova@catalysis.ru (M.A.); valeria@catalysis.ru (V.F.); valeev@catalysis.ru (K.V.); yeremeev21@gmail.com (N.E.); sadovsk@catalysis.ru (E.S.); krieger@catalysis.ru (T.K.); glazn@catalysis.ru (T.G.); sadykov@catalysis.ru (V.S.); smike@catalysis.ru (M.S.)

**Keywords:** methane dry reforming, supercritical synthesis, fluorite, ceria–titania, heterogeneous catalysis

## Abstract

A series of 5%Ni/Ce_1-x_Ti_x_O_2_ catalysts was prepared with nickel impregnation of mixed Ce–Ti oxides obtained via synthesis in supercritical isopropanol. All oxides have a cubic fluorite phase structure. Ti is incorporated into the fluorite structure. Small amounts of impurities of TiO_2_ or mixed Ce–Ti oxides appear with Ti introduction. Supported Ni is presented as the NiO or NiTiO_3_ perovskite phase. Ti introduction increases total samples reducibility and results in stronger interaction of supported Ni with the oxide support. The fraction of rapidly replaced oxygen and the average tracer diffusion coefficient also increase. The number of metallic nickel sites decreased with increasing Ti content. All catalysts except Ni-CeTi0.45 demonstrate close activity in tests of dry reforming of methane. The lower activity of Ni-CeTi0.45 can be connected to Ni decoration with species of the oxide support. The incorporation of Ti prevents detachment of Ni particles from the surface and their sintering during dry reforming of methane.

## 1. Introduction

The global growth of energy consumption and environmental pollution by fuel combustion products require development of new approaches to energy production. Dry reforming of methane (DRM) (Equation (1)) has attracted great interest among researchers, since it enables utilizing the greenhouse gases methane and CO_2_ to obtain synthesis gas, which can be used for the synthesis of valuable chemical compounds [1,2].

Stoichiometrically, the DRM reaction can produce synthesis gas with a H_2_:CO ratio of 1:1, which is the most attractive for further use such as production of long-chain hydrocarbons by Fischer–Tropsch synthesis [3,4]:

CH_4_ + CO_2_ ⇄ 2CO + 2H_2_ ΔH^0^_298K_ = 247.3 kJ/mol (1)

However, the occurrence of side reactions can change this ratio. In particular, the reverse water–gas shift reaction leads to its decrease (Equation (2)) [5]:

CO_2_ + H_2_ ⇄ CO + H_2_O ΔH^0^_298K_ = 41.0 kJ/mol (2)

Despite the attractiveness, the DRM reaction is still not used in the industry since the studied catalysts have a number of disadvantages. Catalysts with noble metals are highly active and stable, but too expensive for practical applications [6]. Ni-containing catalysts have an affordable price and also show high activity; however, they are deactivated due to carbon formation and sintering of supported metal particles [7].

The solid carbon can be formed by reactions such as CH_4_ decomposition (Equation (3)) and CO disproportionation (or Boudouard reaction) (Equation (4)) [8]:

CH_4_ ⇄ C + 2H_2_ ΔH^0^_298K_ = 75.0 kJ/mol (3)

2CO ⇄ C + CO_2_ ΔH^0^_298K_ = −172.0 kJ/mol (4)

Since methane cracking is a structure-sensitive reaction and occurs on ensembles of nickel atoms larger than that required for the DRM reaction, a high dispersion of Ni metal particles should prevent carbon formation [9,10,11,12].

One of the ways to obtain high metal dispersion is modification of the active component by addition of another metal and subsequent alloy formation [13,14]. Another approach includes the selection of the catalyst support with optimal characteristics such as textural features, thermal stability, redox properties, oxygen mobility and ability to stabilize supported metal in a highly dispersed state [15,16].

Different types of supports were investigated for DRM catalysts, including Al_2_O_3_, SiO_2_, La_2_O_3_, ZrO_2_, MgO, TiO_2_ and CeO_2_, also modified by various cations [4,7,17]. The most widely used oxide support for DRM catalysts is Al_2_O_3_. However, acidic sites on its surface cause carbon formation, leading to catalyst deactivation. Another possible reason for low activity is the formation of inactive NiAl_2_O_4_ [17,18,19,20]. Catalysts based on inert SiO_2_ show weak metal–support interaction and are deactivated due to metal sintering and carbon formation [18,21]. MgO has a similar crystallite structure to NiO so it can form NiO-MgO solid solution that enables obtaining small nickel metal particles during the reaction. Further, MgO contains basic surface sites that enhance CO_2_ chemisorption, which leads to reduced carbon formation and higher catalytic stability [7,17].

The use of oxides with a high oxygen storage capacity (OSC) in addition to oxygen mobility as supports should prevent catalyst deactivation due to carbon formation, since the support oxygen is involved in the process of coke precursor gasification [4,22]. DRM over catalysts with high oxygen mobility includes the following stages: decomposition of CH_4_ on supported Ni particles with the formation of H_2_ and CH_x_ fragments, activation of CO_2_ on oxygen vacancies in the oxide support with the formation of CO and active lattice oxygen, diffusion of active oxygen from the support to metal particles followed by oxidation of CH_x_ fragments [12,23,24].

Among investigated supports, ceria is known to have high OSC and Ce^4+^/Ce^3+^ redox potential [1,25]. The presence of oxygen vacancies associated with the variable valency of Ce also plays an important role, since they act as sites for CO_2_ activation with the formation of active oxygen species and re-oxidation of the reduced oxide support [26,27]. In addition, ceria prevents sintering of the active component due to the strong metal–support interaction providing a high dispersion of Ni particles [1].

However, CeO_2_ is susceptible to sintering at high temperatures, which leads to a decrease in OSC [28]. The modification of CeO_2_ with Zr, La and Pr cations results in increasing OSC, reducibility, thermal stability and improves textural characteristics [29,30,31,32].

Catalysts based on TiO_2_ also demonstrated high stability in the DRM reaction due to the ability of TiO_x_ species to decorate particles of supported metal during reduction treatment, which leads to the dilution of ensembles of nickel atoms and prevent carbon formation. In addition, oxygen vacancies on the surface of reduced TiO_2_ can also serve as activation sites for carbon dioxide [33,34].

Therefore, it is interesting to dope CeO_2_ by titanium to improve its characteristics. In addition, the substitution of rare earth cerium with more affordable and inexpensive titanium makes sense in terms of economic benefits.

In the work of Luo et al. [28] it was shown that doping of CeO_2_ with titanium also leads to the increased reducibility of mixed oxides in comparison with pure ceria. Samples with a titanium content of 0.1 to 0.4 represented a single-phase fluorite structure according to XRD data.

The EXAFS studies and DFT calculations confirmed that titanium introduction into CeO_2_ leads to distortion of its crystal structure, resulting in the formation of longer and shorter Ce-O and Ti-O bonds than the Ce-O bonds in the initial oxide. The longer Ce-O bonds and all Ti-O bonds are weaker than in the initial ceria, which results in the increased OSC [35].

The utilization of mixed Ce–Ti oxides as catalysts supports enables improving their catalytic activity in various reforming reactions [26,36,37,38].

A series of 10%Ni/CeO_2_-TiO_2_ catalysts were investigated in dry reforming of methane in the work [36]. It was shown that catalysts containing Ti in the amount of 20 and 50 at.% provided higher activity than pure Ni/CeO_2_ and higher activity and stability than pure Ni/TiO_2_.

Studies in the DRM by Damaskinos et al. [26] showed that catalysts based on Ce_0.8_Ti_0.2_O_2_ and Ce_0.5_Ti_0.5_O_2_ provided close activity, but Ce_0.8_Ti_0.2_O_2_ was significantly less prone to carbon formation compared to Ce_0.5_Ti_0.5_O_2_ and CeO_2_.

The activity of 10%NiO/Ce_x_Ti_1-x_O_2_ catalysts series was investigated in the reactions of partial oxidation of methane [37] and steam reforming of ethanol [38]. The catalyst based on the Ce_0.5_Ti_0.5_O_2_ oxide support provided the highest activity in both reactions. The supported Ni was presented in the form of NiTiO_3_ perovskite for Ti-containing catalysts, and the authors assumed that this phase plays a key role in the high catalytic activity.

Thus, the introduction of titanium into CeO_2_ leads to modification of both the oxide support and the supported active component, which is of great interest for further studies of mixed Ce–Ti oxides as catalyst supports.

It is well known that the preparation method has a great influence on the properties of the resulting oxides. For the preparation of mixed ceria–titania oxides, various methods were used: the sol-gel method [28], solution combustion method [35], mixing in a mortar [36] and co-precipitation [37,38].

Earlier, we showed that synthesis in supercritical isopropanol in a continuous-flow installation enables obtaining a single-phase mixed Ce–Zr oxide without impurity of the ZrO_2_ phase, which was observed in oxides prepared by the citrate method [39]. Moreover, such a preparation method results in the formation of a higher amount of oxygen vacancies. Synthesis in supercritical alcohols is also characterized by low energy consumption and affects the morphology of resulting oxides by varying synthesis conditions such as pressure and temperature [40,41]. Therefore, this method was chosen for the synthesis of mixed ceria–titania oxides.

The main goal of this work was to investigate the influence of Ti introduction on the properties of mixed Ce–Ti oxides and catalytic activity in dry reforming of methane. The novelty of this work includes the following aspects: the preparation of a series of mixed Ce–Ti oxides by continuous-flow synthesis in supercritical alcohol, investigation of their physicochemical properties and the catalytic activity of Ni-containing catalysts in the DRM reaction, and study of the influence of Ni addition on the oxygen mobility of the samples.

## 2. Results and Discussion

### 2.1. Characterization of the Textural Properties of the Samples by N_2_ Adsorption

The compositions of mixed oxides, catalysts and their textural characteristics are presented in Table 1. The specific surface area of individual CeO_2_ is 35 m^2^/g and it decreases with titanium introduction from 26 to 17 m^2^/g.

**Figure 1:**
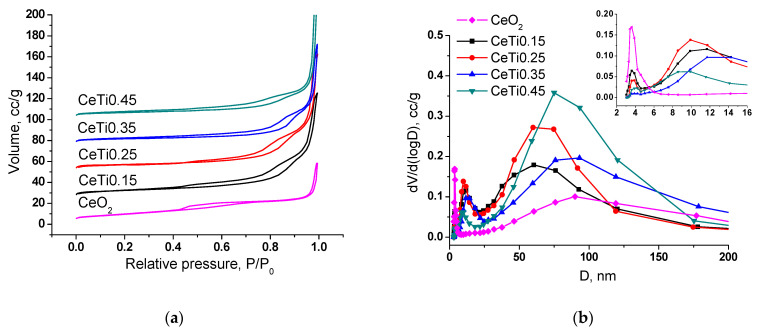
Nitrogen adsorption–desorption isotherms (**a**) and pore size distribution (**b**) for the mixed Ce_1-x_Ti_x_O_2_ oxides.

Nitrogen adsorption–desorption isotherms on mixed Ce–Ti oxides are characteristic for materials with a mesoporous structure (IV type) (Figure 1a). The capillary condensation hysteresis loop is assigned to the condensation of adsorbate in mesopores [42]. The pore volume increases with Ti introduction almost 2 fold from 0.085 for CeO_2_ to 0.157 cm^3^/g for CeTi0.15, but no further correlation with titanium content is observed in the series of mixed oxides.

The samples are characterized by a polymodal pore size distribution presented in Figure 1b. There are a few micropores (maximum distribution at 4 nm), mesopores (10–12 nm, except CeO_2_) and macropores (60–93 nm). No mesopores are observed for CeO_2_, which explains its lower pore volume and the different isotherm shape (type I, microporous solids). It should be noted that the micropore volume significantly decreases with titanium introduction, probably resulting in a decreased surface area of the samples.

After nickel deposition, the specific surface area decreases by 5–10 m^2^/g due to partial blocking of micro- and mesopores by NiO particles, while the total pore volume slightly increases.

### 2.2. Characterization of the Structural Features of the Samples by XRD and Raman Spectroscopy

**Figure 2:**
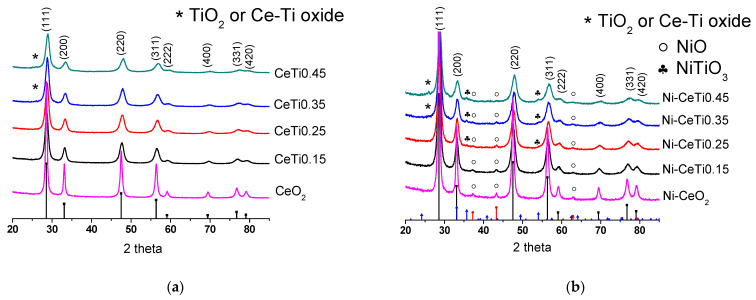
Diffraction patterns of mixed oxides (**a**) and catalysts (**b**), calcined at 700 °C. Reference patterns correspond to: black + (hkl) indices—CeO_2_ [PDF 00-043-1002], red—NiO [PDF 00-044-1159], and blue—NiTiO_3_ [PDF 04-010-7290].

Figure 2 presents diffractograms of mixed oxides and catalysts with supported 5 wt.% nickel. The pure CeO_2_ has a cubic fluorite structure [PDF 00-043-1002] with a cell parameter of 5.411 Å (Table 1). After titanium introduction, the fluorite structure is retained. The shift in reflections towards large angles occurs due to a decrease in lattice parameter. The lattice parameter decreases with increasing Ti content from 5.400 to 5.388 Å due to Ti incorporation into the ceria structure and the smaller ionic radius of titanium, Ti^4+^ (0.74 Å) < Ce^4+^ (0.97 Å) [43].

The fluorite phase is only observed at a titanium content of 0.15–0.25. In samples with a titanium content of 0.35–0.45, a weak broad shoulder appears in the region of 26°, which can be attributed to TiO_2_ with a brookite structure [PDF 003-0380] or to mixed Ce–Ti oxide, Ce_0.66_TiO_2.975_ [PDF 033-0342]. The crystallite size of the fluorite phase is significantly reduced with titanium introduction from 29 nm for pure CeO_2_ to 11–13 nm for mixed Ce–Ti oxides, and it continues to decrease slightly with increasing titanium content. Such a decrease may be due to the amorphous titanium oxide phase, which prevents sintering [44].

Supported nickel is presented as the NiO phase [PDF 00-044-1159] for catalysts with a low titanium content (Figure 2b). With increasing Ti content in the support, peaks of the NiTiO_3_ perovskite phase [PDF 04-010-7290] appear while the intensity of NiO peaks gradually decreases. For the Ni-CeTi0.45 catalyst with the highest Ti content, only reflections of the perovskite phase are observed. The cell parameter of the fluorite phase practically does not change after nickel deposition, which probably indicates that nickel is not incorporated into the support structure. However, as will be shown below, it can be connected to method limitations and Ni can be incorporated in minor quantities or into surface layers. The crystallite size slightly increased, possibly due to an additional calcination step after nickel deposition.

**Figure 3:**
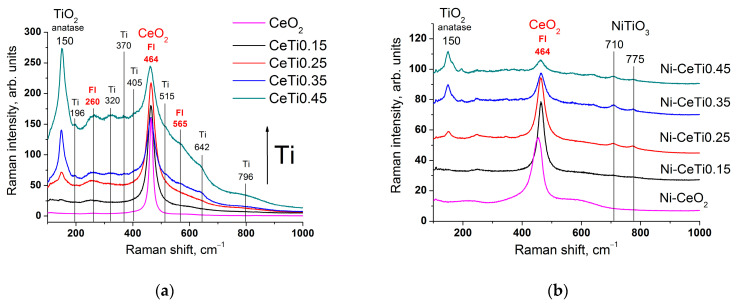
Raman spectra of mixed oxides (**a**) and catalysts (**b**), calcined at 700 °C. Catalyst spectra are separated by height for the convenience of readers.

The structure of the samples was further investigated using Raman spectroscopy, which characterizes the vibrations of the oxygen lattice and is more sensitive to crystalline symmetry and the microstructure of materials. For all mixed oxides, a high-intensity band is observed in the region of 464 cm^−1^, which corresponds to the fundamental F_2g_ symmetry mode of the cubic fluorite structure and is attributed to a symmetrical stretching mode of the Ce–O8 vibrational unit [45] (Figure 3a). After titanium introduction, the fluorite band broadens, which indicates the increased dispersion of its particles [46] and is in agreement with XRD data. Weak broad bands at 260 and 565 cm^−1^ can be assigned to the transverse acoustic (TA) mode and the non-degenerate longitudinal optical (LO) mode due to the presence of defects, which results from the formation of oxygen vacancies in the fluorite structure due to the substitution of Ce^4+^ by an ion with a different radius [47,48,49].

The introduction of Ti also leads to the appearance of a high-intensity band at 150 cm^−1^ (E_g_ mode), corresponding to TiO_2_ in the anatase phase. Weaker bands at 196 (E_g_), 405 (B_1g_), 515 (A_1g_ or B_1g_), 642 (E_g_) and 796 (overtone of B_1g_) cm^−1^ also refer to the anatase structure [50,51]. With increasing titanium content, the intensity of the anatase bands and the overall absorption background increase, suggesting that TiO_2_ is in a highly dispersed state. The position of the main anatase band is shifted to higher wavenumbers compared to a single crystal value (150 instead of 144 cm^−1^), which is also related to the high dispersion of its particles [50]. Weak bands at 320 and 370 cm^−1^ can be attributed to certain defects in TiO_2_ structure due to its nanocrystallinity [52].

The total spectra intensity significantly decreases after nickel deposition due to the absorption of radiation by nickel. For Ni-CeO_2_ composition, the main fluorite band is shifted to lower wavenumbers (453 instead of 464 cm^−1^) and the intensity of bands assigned to oxygen vacancies in the fluorite structure increases, which can be due to the incorporation of Ni into the ceria lattice [47,53]. For other catalysts, the main fluorite band retains its position. For samples with a high Ti content, there are bands at 710 and 775 cm^−1^, which both correspond to the A_g_ mode of the NiTiO_3_ phase [54,55].

### 2.3. Characterization of the Morphology of Fresh Catalysts by HRTEM

The morphology of fresh catalysts based on pure CeO_2_ and mixed oxides with the lowest and the highest Ti content was investigated by HRTEM and HAADF-STEM with EDX analysis. Synthesis in supercritical alcohols results in the formation of oxide particles with close to spherical morphology, with sizes of approximately 15–20 nm for Ni-CeO_2_ and 5–10 nm for Ni-CeTi, which is close to XRD data. They are packed into large spherical aggregates with a diameter greater than 100 nm. A similar morphology was determined in our earlier works for catalysts based on ceria-zirconia that were also prepared in supercritical media [39,56].

**Figure 4:**
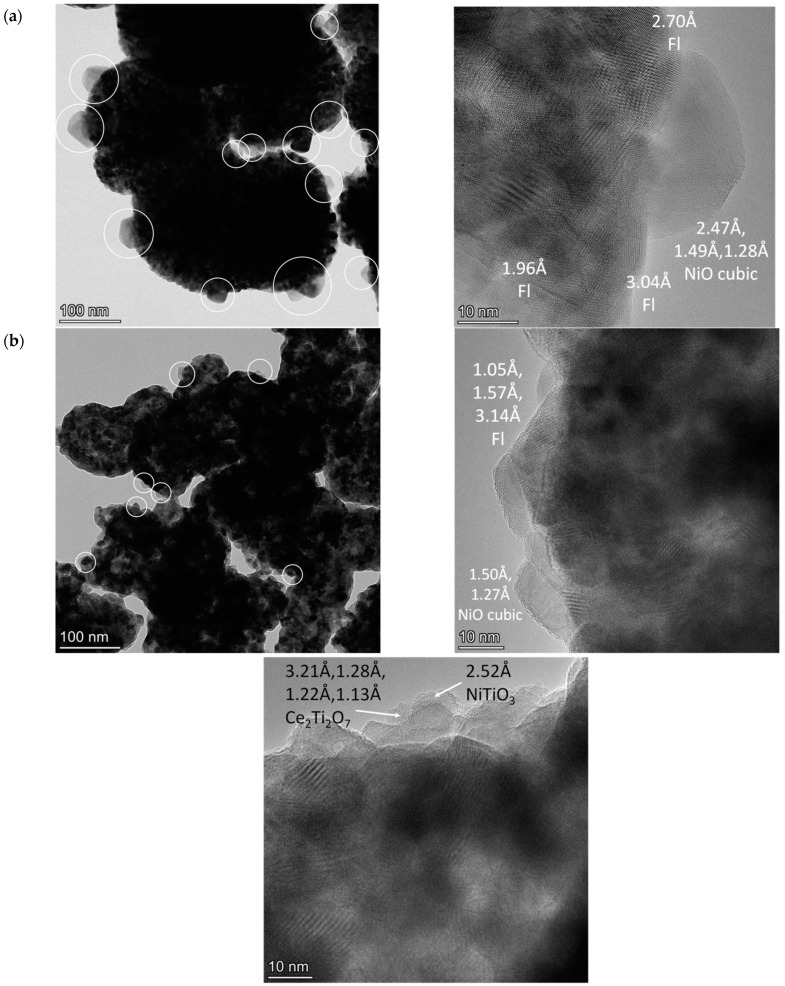

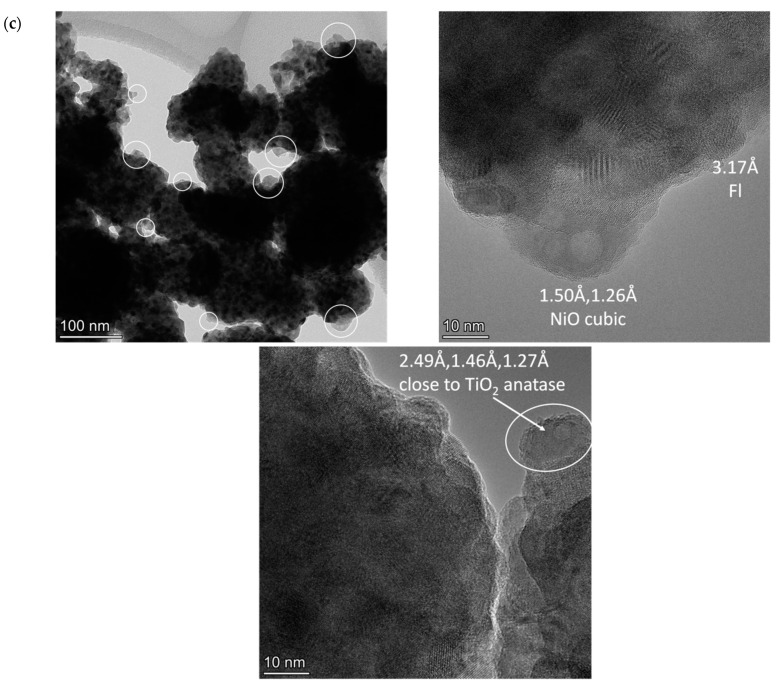
HRTEM images of fresh catalysts, 5%Ni/Ce_1-x_Ti_x_O_2_, calcined at 700 °C: (**a**) Ni-CeO_2_, (**b**) Ni-CeTi0.15, and (**c**) Ni-CeTi0.45. The particles of nickel oxide are marked by circles. Fl—fluorite.

For catalysts based on pure ceria, NiO particles have an almost cubic shape, with particles sizes of 25–50 nm (Figure 4a). For Ti-containing catalysts, NiO particles have a more flattened shape, suggesting a stronger metal–support interaction and crystallite sizes of approximately 10–20 nm (Figure 4b,c).

For titanium-doped catalysts, in addition to particles of NiO and fluorite, there are finely dispersed particles of TiO_2_, mixed oxide, Ce_2_Ti_2_O_7_, and perovskite, NiTiO_3_, on the surface of fluorite. Under an electron beam in a microscope column, titanium oxide often changes its morphology associated with the simultaneous destruction of the near-surface layer and the generation of island formations.

It should be noted that perovskite particles are already present in the Ni-CeTi0.15 composition, while NiO particles are observed in the Ni-CeTi0.45 sample, although no such peaks are observed in XRD data—probably due to small amount of the corresponding phases.

**Figure 5:**
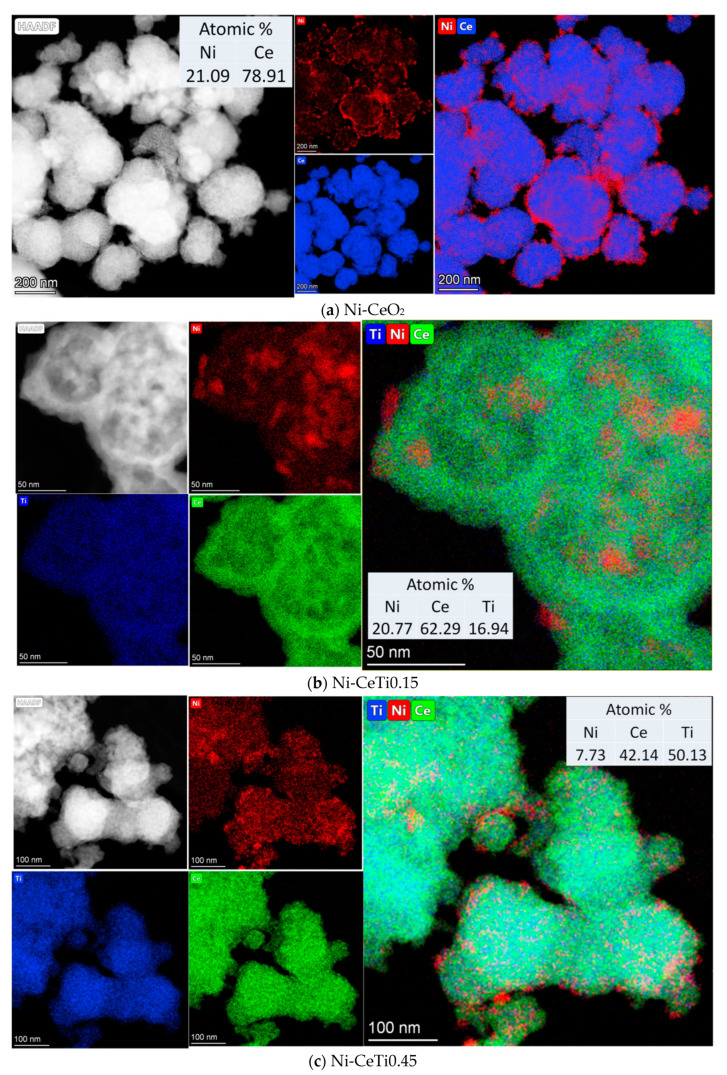
HAADF-STEM image with EDX analysis of fresh catalysts, 5%Ni/Ce_1-x_Ti_x_O_2_, calcined at 700 °C: (**a**) Ni-CeO_2_, (**b**) Ni-CeTi0.15, and (**c**) Ni-CeTi0.45.

The dark-field images with EDX analysis of Ni-CeO_2_ demonstrated that nickel is distributed in the form of large particles over the surface of fluorite particle agglomerates (Figure 5a). For Ti-doped catalysts, the homogeneous distribution of Ti and Ce cations in the oxide structure is observed even at high Ti contents and Ni distribution is more uniform than for pure CeO_2_, suggesting that some Ni cations can be incorporated into the fluorite lattice.

### 2.4. Characterization of the Surface Sites of Catalysts by FTIR Spectroscopy of Adsorbed CO

**Figure 6:**
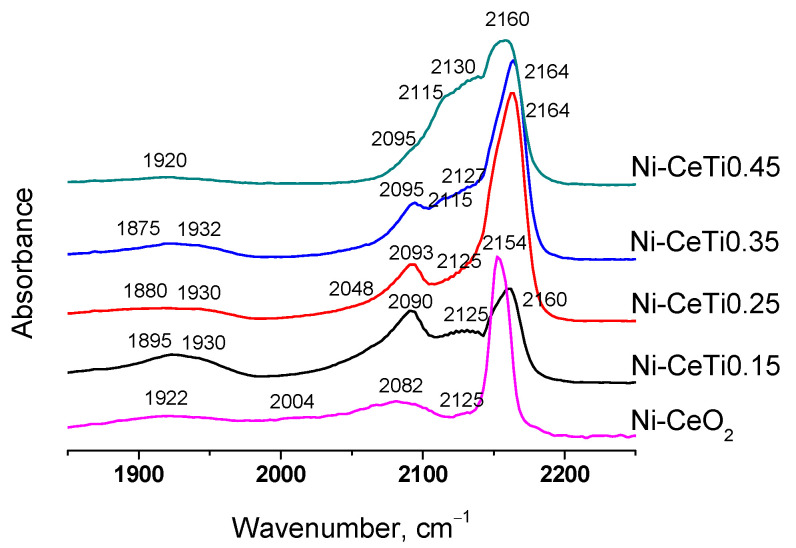
Differential FTIR spectra of CO adsorbed on catalysts at −196 °C, p_CO_ = 10 Torr.

The nature of the surface sites of the catalysts was investigated using FTIR spectroscopy of adsorbed CO. The catalysts were preliminary reduced in hydrogen atmosphere at 600 °C for 1 h. Figure 6 presents the differential spectra of CO adsorbed on catalysts at −196 °C and p_CO_ = 10 Torr.

Adsorption bands at 2154–2164 cm^−1^ correspond to CO adsorption on support cations Ce^4+^ and Ti^4+^ [57,58,59,60,61]. For undoped Ni-CeO_2_, the adsorption band at 2154 cm^−1^ is symmetric and assigned to CO adsorption on Ce^4+^ cations. With Ti introduction, the band of CO complexes with Ti^4+^ cations appears at 2160–2164 cm^−1^, due to which the band at 2154 cm^−1^ is slightly shifted and becomes asymmetric. In addition, the shift towards higher frequencies can be due to the increase in the adsorption site strength of Ce^4+^ upon introduction of doping cations [39].

It should be noted that there are no bands at 2180–2200 cm^−1^ corresponding to CO complexes with strong Lewis acid sites of Ti^4+^ which are usually observed for TiO_2_ or Ti-containing materials [59,62]. Their absence can indicate that metal Ni particles interact with these Ti sites [59].

The bands at 2120–2130 and 2110–2120 cm^−1^ correspond to CO complexes with reduced support cations Ce^3+^ and Ti^3+^, respectively [58,59,60]. The band of Ce^3+^-CO complexes is observed for all samples and its intensity increased with Ti introduction, suggesting the increased reducibility of mixed Ce–Ti oxides. The band of Ti^3+^-CO complexes appeared for samples with a high Ti content.

The low-frequency bands at 2004–2095 and 1875–1932 cm^−1^ are assigned to terminal (on-top) and bridging CO complexes with Ni^0^ sites [63,64].

Based on the intensity of these bands, the number of various types of surface carbonyls was estimated (Table 2). The total amount of Ni^0^ sites was shown to decrease with Ti content. The same trend was observed by XPS data in the literature [36]. Such an effect might be due to incomplete nickel reduction or partial decoration of Ni particles with support species.

The tendency of TiO_2_ support to decorate supported metal particles during reduction was shown in the literature [65,66]. Moreover, this effect was observed at lower reduction temperatures (>500 °C) compared with ceria-based catalysts (>700 °C) [65].

### 2.5. Characterization of Sample Reducibility by TPR-H_2_

**Figure 7:**
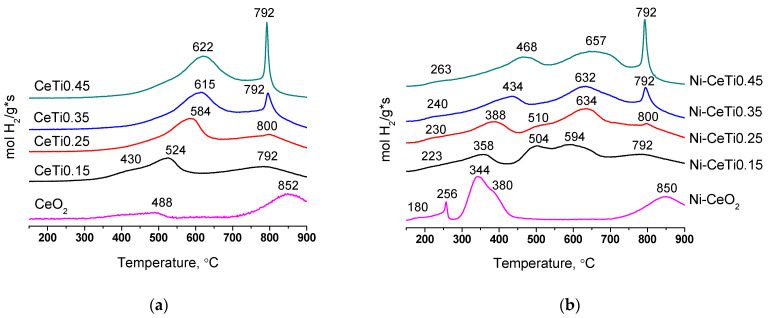
TPR profiles of mixed oxides (**a**) and catalysts (**b**), calcined at 700 °C.

The reducibility of mixed oxides and catalysts was investigated by TPR-H_2_. Reduction profiles of mixed oxides are presented in Figure 7a. For pure CeO_2_, there are two reduction peaks—one peak with low-intensity at 488 °C and a high-intensity peak at 852 °C—which correspond to the reduction of oxygen surface species and the bulk oxygen of CeO_2_, respectively [28,67].

The amount of hydrogen consumed for CeO_2_ sample reduction is 0.82 mmol of H_2_/g oxide (Table 3), and the resulting formula can be written as CeO_1.86_. (oxidation state of Ce is +3.72). With the increase in Ti content, reduction depth increases. For sample with the highest titanium content, 1.77 mmol of H_2_/g oxide is consumed and it can be estimated that the mixed oxide is reduced to the composition Ce_0.55_Ti_0.45_O_1.77_ (oxidation state of Ce is +3.16 assuming that Ti^4+^ is not reduced). However, it should be noted that this formula is conditional, since the sample also contains an admixture of titanium oxide.

The increase in the total amount of consumed hydrogen with Ti introduction can be explained by the weakening of some Ce-O bonds and weaker Ti-O bonds in the mixed oxide compared to the initial CeO_2_ that results in increased OSC [35]. The behavior of the high-temperature peak, which corresponds to the reduction of Ce^4+^ to Ce^3+^ in the oxide bulk, also correlates with this explanation. With the introduction of titanium, its intensity increases and it shifts to lower temperatures compared to pure CeO_2_.

However, the medium-temperature reduction peak shifts to higher temperatures with increasing titanium content. Similar behavior has also been observed in the literature [28,35,44]. It can be connected to the decrease in the specific surface area with Ti introduction, leading to its reduced availability for hydrogen, which can complicate the reduction of oxygen surface species [29,68].

A larger amount of consumed hydrogen for Ti-doped oxides can also be associated with the reduction of titanium cations from Ti^4+^ to Ti^3+^, the presence of which was confirmed by FTIR spectroscopy of CO for reduced catalysts. There are ambiguous data about the reduction of TiO_2_ in the literature. In several works, there is no hydrogen consumption for individual TiO_2_ [28,37,44]. In other works, pure TiO_2_ is reduced by hydrogen at temperatures of approximately 450–600 °C [35,59,67]. TiO_2_ doped by Ce has reduction peaks at lower temperatures, from 400 °C [59].

Reduction profiles of catalysts with supported Ni are presented in Figure 7b. Nickel deposition affects the nature of reduction in the range of low and medium temperatures, while the high-temperature peak for all catalysts remains unchanged and retains its position and intensity. This means that nickel only has an impact on the reduction of oxides surface, but not the bulk of oxides. Therefore, below, we will focus on low- and medium-temperature regions.

The deposition of nickel contributes to lowering the temperature of reduction beginning at 200 °C since the presence of NiO facilitates the reduction of active oxygen surface species [38,69]. Peaks at 220–260 °C can be attributed to the reduction of oxygen adsorbed on the surface. The introduction of nickel into the CeO_2_ structure leads to the formation of oxygen vacancies, which can absorb oxygen, being easily reduced at such low temperatures [38,69].

It should be noted that no change in the fluorite lattice parameter is observed according to XRD data. However, for Ni-CeO_2_ sample, there is a shift in the main fluorite band in the Raman spectrum, which may indicate the partial incorporation of nickel cations into the oxide structure. In addition, the incorporation of Ni only into the surface layers may be not visible from XRD data.

Peaks at 344 and 380 °C for the Ni-CeO_2_ sample correspond to the reduction of NiO, weakly interacting with the oxide support jointly with Ce^4+^ cations in the surface layers [36,38].

For other catalysts, the joint incorporation of both Ti and Ni cations into fluorite surface leads to a more complex picture. Generally, these TPR profiles have a typical shape for impregnated nickel-containing catalysts based on ceria-zirconia, as was shown earlier in our works [39,40]. For these catalysts, there are several reduction peaks from 300 to 700 °C, corresponding to the reduction of various forms of nickel jointly with oxide support cations [29,38,40,70].

With the increase in titanium content, peaks shift towards higher temperatures, suggesting a stronger interaction of supported Ni with the oxide support. For samples with a titanium content of 0.15–0.25, there are two peaks at temperatures of 500–630 °C, which may be due to the simultaneous presence of Ni in the form of nickel oxide NiO and perovskite NiTiO_3_.

It is known that the reduction of nickel from NiTiO_3_ occurs at temperatures of 640–650 °C [36,38]. For samples with a higher Ti content, only one peak is observed at 630–650 °C, suggesting that supported Ni is presented in the form of NiTiO_3_.

However, it should be noted that for the Ni-CeTi0.45 sample, the amount of hydrogen consumed within the peak at 657 °C (1.5 mmol of H_2_/g) is almost 2-fold higher than the amount required for the reduction of all nickel in the catalyst (0.85 mmol H_2_/g), suggesting the joint reduction of Ni and support cations.

Based on the data of hydrogen consumption by the supports and catalysts, the degree of the reduction of supported nickel was calculated using the following formula:

$$\mathrm{Reducibility}=\frac{H_{2}\left(\mathrm{catalyst}\right)-H_{2}\left(\mathrm{support}\right)\cdot 0.95}{0.85}\cdot 100\%$$

where H_2_ (catalyst) and H_2_ (support) [mmol H_2_/g]—amount of hydrogen consumed for catalyst and support reduction, respectively; 0.95—support content in the catalyst; 0.85 [mmol H_2_/g]—theoretical amount of H_2_ needed to reduce all supported Ni.

The calculated value turned out to be close for all samples (from 80 to 88%) and no obvious correlation with titanium content was observed. Ni reducibility is less than 100%, which can be explained by the incorporation of minor quantities of Ni into the fluorite lattice which are not reduced during TPR tests.

### 2.6. Characterization of the Oxygen Mobility of the Samples by Isotope Exchange with C^18^O_2_

**Figure 8:**
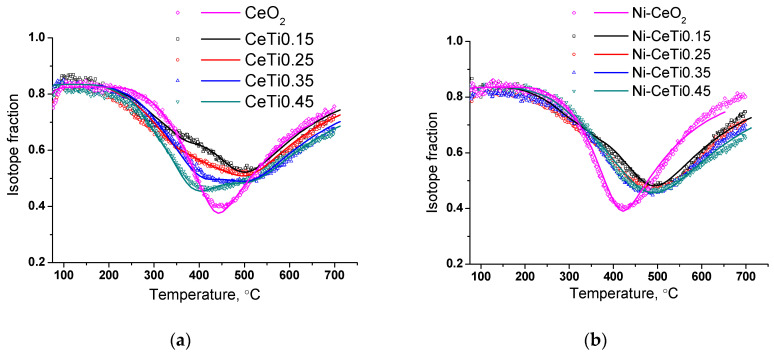
TPIE curves for mixed oxides (**a**) and catalysts (**b**). Points—experiment; lines—modeling.

Experiments of the temperature-programmed isotope exchange (TPIE) of oxygen with C^18^O_2_ in a flow reactor were carried out to investigate the influence of Ti incorporation on oxygen transport characteristics. The experimental and simulated isotope fraction curves for supports and catalysts are presented in Figure 8. The diffusion activation energy for all samples is approximately 150 kJ/mol.

For pure CeO_2_, the oxygen substitution rate is satisfactorily described by a homogeneous diffusion model which corresponds to the presence of one type of oxygen in its composition and is consistent with XRD data. The calculated oxygen tracer diffusion coefficient at 600 °C is approximately 2.2 × 10^−13^ cm^2^/s (Table 4). 

With Ti introduction, the exchange starts at a lower temperature compared to that for undoped CeO_2_. Moreover, there are several overlapping peaks on TPIE curves, which indicate the presence of various forms of oxygen. Most of the oxygen is substituted at approximately the same rate as for CeO_2_, with a diffusion coefficient of 2.2 × 10^−13^ cm^2^/s. At the same time, there are oxygen species which are substituted by an order of magnitude faster, with a diffusion coefficient of 3 × 10^−12^ cm^2^/s. With the increase in Ti content, the amount of rapidly substituted oxygen increases from 10% for CeTi0.15 to 30% for CeTi0.45.

This fact is consistent with the above considerations about the formation of weaker Ce-O and Ti-O bonds after titanium incorporation [35]. Thus, the average diffusion coefficient increases with Ti introduction from 2.2 × 10^−13^ cm^2^/s for CeO_2_ to 11 × 10^−13^ cm^2^/s for CeTi0.45.

Nickel addition has a complex effect on the nature of exchange. For undoped Ni-CeO_2_, the exchange rate increases and this may be due to the partial incorporation of nickel cations into the fluorite structure, resulting in lattice disordering and the formation of oxygen vacancies. For samples doped with titanium, 5%Ni/Ce_1-x_Ti_x_O_2_, the exchange rate decreases and the isotopic fraction curves become more uniform, which can be explained by two phenomena. Firstly, the amount of rapidly substituted oxygen is reduced to 3–5%. Such an effect might be associated with decrease in the oxygen permeability of near-surface layers in the mixed oxide after nickel supporting and was previously shown for the ceria-zirconia catalysts [39]. Secondly, with the increase in Ti content the exchange rate of the main oxygen forms increases to a lower extent since most of the nickel cations are in the form of perovskite NiTiO_3_ and are not incorporated into the fluorite lattice.

Thus, the average oxygen tracer diffusion coefficient for catalysts decreases with increasing Ti content due to decrease in the amount of rapidly substituted oxygen and the smaller increase in the exchange rate of the remaining oxygen.

### 2.7. Catalytic Studies in Dry Reforming of Methane

**Figure 9:**
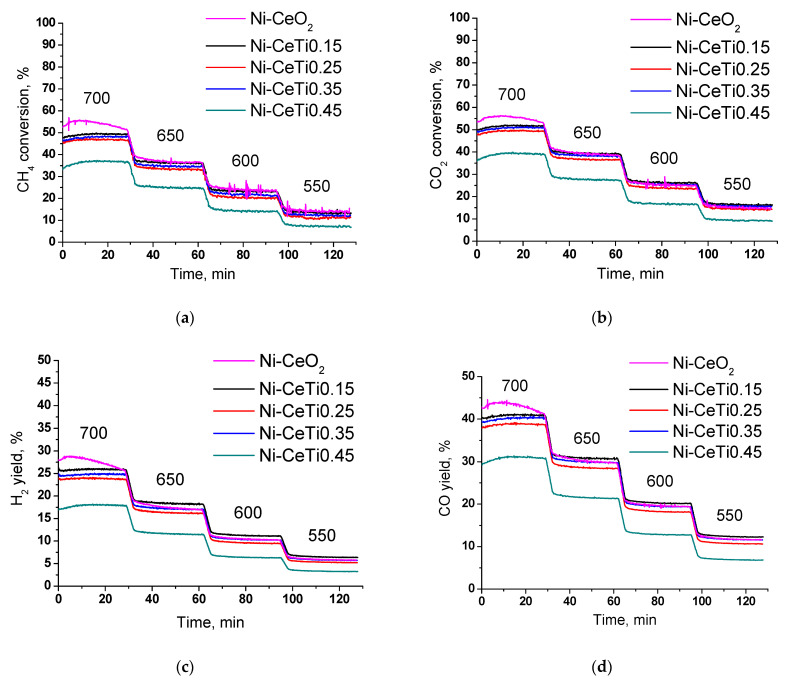

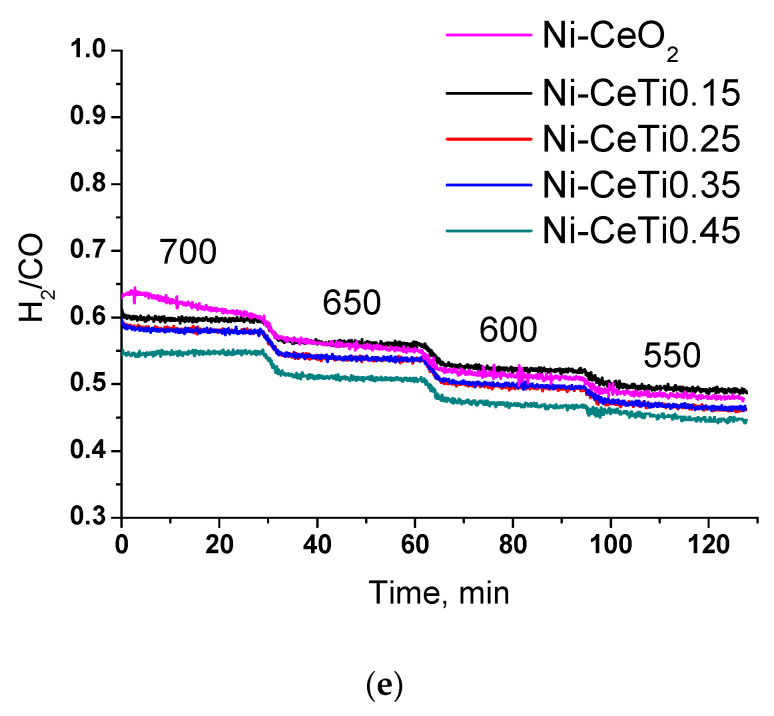
Results of temperature catalytic tests in dry reforming of methane: (**a**) CH_4_ conversion, (**b**) CO_2_ conversion, (**c**) H_2_ yield, (**d**) CO yield, (**e**) H_2_/CO ratio, 15% CH_4_ + 15% CO_2_ + N_2_, τ = 10 ms.

Figure 9 presents the results of catalytic tests of 5%Ni/Ce_1-x_Ti_x_O_2_ in dry reforming of methane at temperatures of 550–700 °C. The reaction was started at 700 °C and then the temperature was gradually lowered. The reagents (CH_4_ and CO_2_) conversions and the products (H_2_ and CO) yields decrease with lower temperature due to reaction endothermicity. The conversion of CO_2_ is slightly higher than that of CH_4_ for the same catalyst composition due to reverse the water–gas shift reaction (Equation (2)). The exception is the Ni-CeO_2_ catalyst, for which conversions of methane and CO_2_ were almost equal at the beginning of the reaction and at 550 °C.

The activity of all catalysts except Ni-CeTi0.45 is close. Methane conversion at the end of 700 °C is in the range 47–52%, with the highest conversion observed for the undoped Ni-CeO_2_. For sample with the highest Ti content, conversion is less by 10% and is equal to 37%. With decreasing temperature, the difference in conversions becomes less significant and amounts to only 3% at 550 °C. The same trend is observed for values of hydrogen yield—the highest value at 700 °C is 27% for Ni-CeO_2_ and the lowest one is 18% for Ni-CeTi0.45.

**Figure 10:**
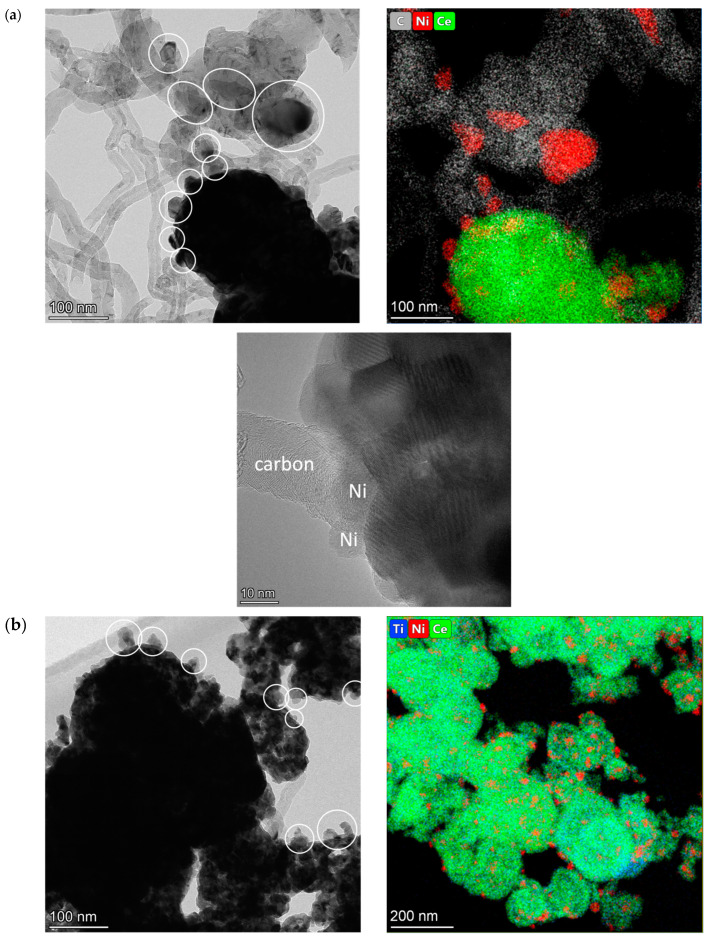

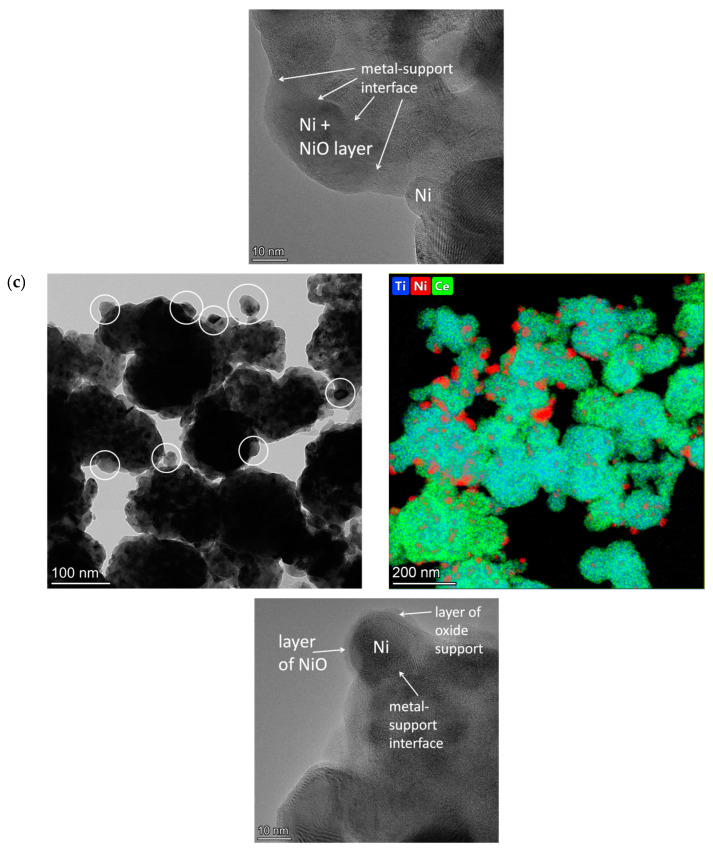
HRTEM and HAADF-STEM images of catalysts, 5%Ni/Ce_1-x_Ti_x_O_2_ after catalytic tests in DRM: (**a**) Ni-CeO_2_, (**b**) Ni-CeTi0.15, and (**c**) Ni-CeTi0.45. The particles of metallic Ni are marked by circles.

However, the catalytic performance of Ni-CeO_2_ at 700 °C differs from the other titanium-containing catalysts. A significant increase in conversion followed by its decrease is observed within the 30 min of the reaction. The H_2_/CO ratio changes in the same way. According to microscopy after the reaction (Figure 10a), there are a lot of carbon fibers formed in the undoped Ni-CeO_2_. Moreover, a lot of Ni particles are detached from the support surface due to the growth mechanism of carbon fibers [71,72]. As was mentioned above (Section 2.3), large particles of NiO in weak contact with the support surface were present in the fresh Ni-CeO_2_ catalyst. Based on the available data, it can be assumed that the methane cracking reaction proceeds on large nickel particles to a great extent with the formation of hydrogen and carbon at the beginning of the reaction. This assumption is also confirmed by the fact that methane conversion is equal to CO_2_ conversion. As a result, the active sites necessary for methane decomposition are blocked by the produced carbon and then DRM becomes the main reaction, as for other catalysts. Since no noticeable activity decrease is subsequently observed for this sample, it can be supposed that a steady state between the reactions of carbon formation and oxidation is reached [71].

The activity of the Ni-CeTi0.45 sample is lower than that of other catalysts over the entire temperature range. These are actually unexpected results, since there is no significant difference between catalysts with titanium contents of 0.35 and 0.45 according to the data of physicochemical methods. Such a difference in activity can be explained by Ni decoration with species of the titanium-containing oxide support, which results in its inaccessibility for the reaction mixture.

The HRTEM images of Ti-containing catalysts are presented in Figure 10b,c. There are almost no carbon fibers in both catalysts with the lowest and the highest titanium content. For both catalysts, clear metal–support interfaces are observed and Ni particles are still attached to the surface due to strong metal–support interaction, which is consistent with HRTEM data for fresh catalysts and results of TPR studies. For the Ni-CeTi0.45 sample, there is a partial decoration of metallic nickel with an oxide support layer, which is consistent with the above assumptions and this is possibly the reason for its lower activity. Both these evidences are manifestations of the classical phenomenon of strong metal–support interaction [66,73].

**Figure 11:**
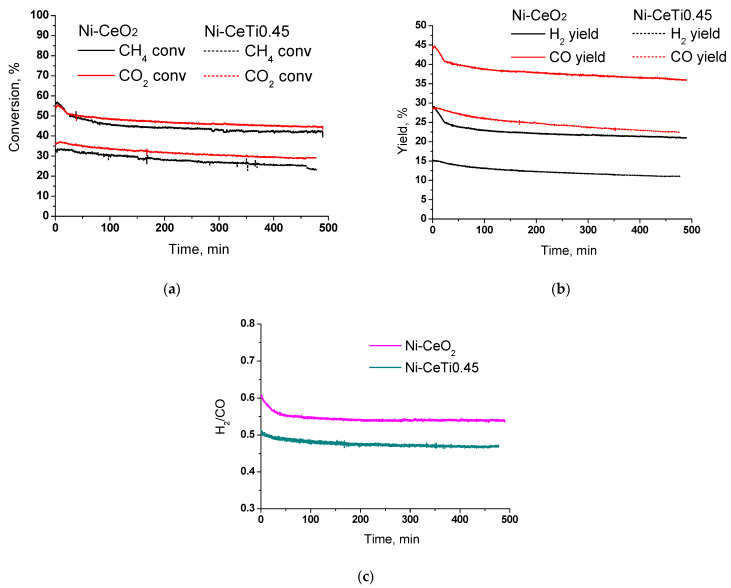
Results of stability catalytic tests in dry reforming of methane: (**a**) reagents conversions, (**b**) products yields, (**c**) H_2_/CO ratio, 15% CH_4_ + 15% CO_2_ + N_2_, τ = 10 ms, T = 700 °C.

In order to study the stability of catalysts, the samples that showed the maximum and minimum activity were tested in DRM in the same reaction mixture at 700 °C for 8 h (Figure 11). At the initial stage, reagents conversions and products yields were similar to those in the temperature tests (Figure 9). For the Ni-CeO_2_ sample, methane conversion was higher than CO_2_ conversion in the first 20 min of the reaction, which may indicate the greater contribution of the methane cracking reaction. Despite different activity values, the samples demonstrated close stability.

After reaching the steady state (30 min of the reaction), the methane conversion for both catalysts was decreased by 8% after 7.5 h—from 50 to 42% for Ni-CeO_2_ and from 33 to 25% for Ni-CeTi0.45. The blocking of active sites of methane cracking in Ni-CeO_2_ by the formed carbon at the first stage is followed by a certain stabilization of the catalyst activity.

The amount of carbon formed during stability tests was determined by temperature-programmed oxidation, and our results and comparison with data in the literature are presented in Table 5. Data in the literature are very contradictory, since samples of the same composition are characterized by different carbon contents. For example, on a 5% Ni/CeO_2_ catalyst after 50 h at 800 °C, 241 mgC/gcat was formed [74], where as 667 mgC/gcat was formed after 10 h [75]; moreover, after 24 h at a lower temperature of 700 °C, which is more thermodynamically favorable for the carbon formation, only 8.9 mgC/gcat was formed [29]. Therefore, it is impossible to directly compare the results obtained with different initial concentrations and temperatures in different works. Even in the case of close reaction conditions, feed rates and contact times are usually different. Further, the amount of carbon formed is non-linearly dependent on time on stream. However, within the same line of catalysts under similar reaction conditions, comparison is possible.

In the current study, it was found that the Ni-CeO_2_ catalyst contained approximately 24.3 wt.% of carbon, while the Ni-CeTi0.45 catalyst had a carbon content of 6.2 wt.%. This is also in agreement with the above results and confirms that the addition of titanium improves carbonization stability. More active carbon formation at the beginning of the reaction on Ni-CeO_2_ does not lead to a greater decrease in activity during further reaction, since the reactions of carbon formation and oxidation are in steady state.

Furthermore, it correlates with our previous data on ethanol dry reforming [78] and some literature data, where a catalyst active in the target reaction is also active in carbon formation [29,75].

### 2.8. Characterization of Ni-CeTi0.45 and Ni-CeTi0.35 by XRD during In Situ Reduction by H_2_

**Figure 12:**
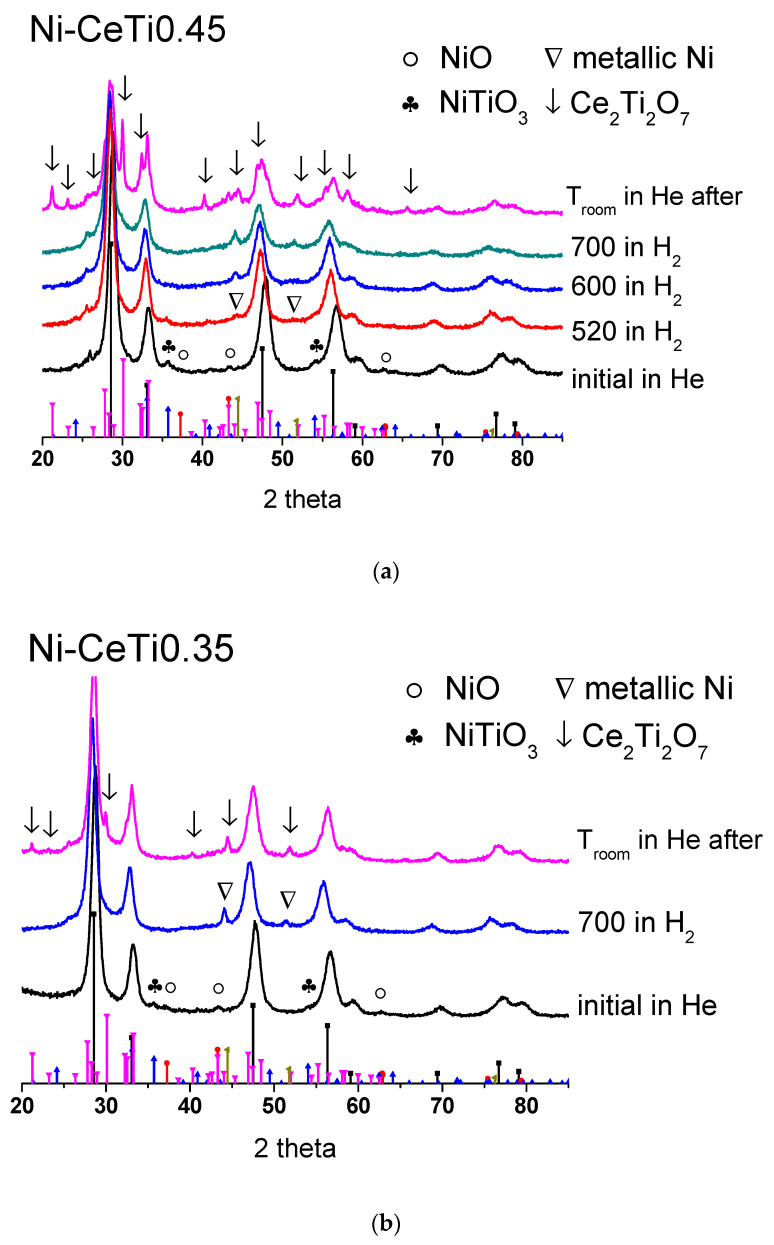
Diffraction patterns of (**a**) Ni-CeTi0.45 and (**b**) Ni-CeTi0.35 recorded during in situ reduction by H_2_. Reference patterns correspond to: black—CeO_2_ [PDF 00-043-1002], red—NiO [PDF 00-044-1159], blue—NiTiO_3_ [PDF 04-010-7290], pink—Ce_2_Ti_2_O_7_ [PDF 00-047-0667], and dark yellow—metallic Ni [PDF 04-010-6148].

According to the results of TPR by H_2_ for the Ni-CeTi0.45 sample, the reduction of support surface layers together with Ni cations is not yet completed at 700 °C, so it was assumed that nickel was not completely reduced during pretreatment before the DRM reaction. To study Ni reducibility, the Ni-CeTi0.45 sample was investigated by XRD during in situ reduction by H_2_. There are peaks of metallic nickel [PDF 04-010-6148] at 520 °C and no other nickel-containing phases are observed at 700 °C, which confirms that most of the nickel was reduced (Figure 12a).

In addition, peaks of the Ce_2_Ti_2_O_7_ oxide [PDF 00-047-0667] appear in the diffraction pattern recorded at room temperature after sample cooling, which indicates the rearrangement of the oxide support. The peaks of the fluorite phase shift towards small angles, which corresponds to the increase in the lattice parameter, probably due to the decrease in the Ti content in the fluorite structure. It is likely that such an rearrangement of the oxide support can also occur during DRM tests, which can lead to decoration of the active component and may explain the lower activity of this composition.

For comparison, the Ni-CeTi0.35 sample was also investigated by XRD during reduction at 700 °C in H_2_ (Figure 12b). After cooling, peaks of the new Ce_2_Ti_2_O_7_ phase also appeared, but with much lower intensity, which indicates its low content.

## 3. Methods and Materials

### 3.1. Catalysts Preparation

The series of mixed Ce_1-x_Ti_x_O_2_ oxides as well as individual CeO_2_ were prepared via solvothermal synthesis in supercritical isopropanol using the continuous-flow mode as was described earlier in [39,56]. The precipitate of the catalyst precursor formed during synthesis was decanted, dried at 200 °C and calcined at 700 °C for 2 h in air.

Nickel was supported by the incipient wetness impregnation of calcined oxides with a water solution of Ni(NO_3_)_2_·6H_2_O (Vecton, pure for analysis) to obtain 5 wt.% Ni content in the catalyst. After impregnation, the samples were dried and calcined at 700 °C for 2 h.

### 3.2. Catalysts Characterization

Experiments of the low-temperature adsorption/desorption of nitrogen were carried out using a Quadrasorb evo (Quantachrome Instruments, Boynton Beach, FL, USA) installation. The specific surface area of the samples was determined by the BET method. The desorption branch of the isotherm was used to estimate the pore volumes by the BJH method.

A Bruker D8 Advance diffractometer (Bruker, Germany) with CuKα radiation and a position-sensitive detector LynxEye was used to record XRD patterns with a step of 0.05° in a 2θ scanning range of 10–85°. Parameter calculations were performed by the Rietveld method using Bruker TOPAS software (Topas V.4.2.).

For experiments of in situ reduction in hydrogen, an XRK-900 high-temperature flow chamber was used. The sample was placed in a chamber and purged with He flow. Next, the heating of the chamber was turned on (heating rate 12 °C/min) and the flow of pure H_2_ was fed. The sample was cooled after exposure in hydrogen to ~150 °C, and then H_2_ was changed to He.

The Raman spectrometer T64000 (Horiba Jobin Yvon) with a micro-Raman setup was used to record the Raman spectra. All experimental spectra were collected in the backscattering geometry using the 514.5 nm line of an Ar^+^ laser. The spectral resolution was not worse than 1.5 cm^−1^. The detector was a silicon-based CCD matrix, cooled with liquid nitrogen. The power of the laser beam reaching the sample was 2 mW. The band at 520.5 cm^−1^ of a Si single crystal was used to calibrate the spectrometer.

Transmission electron microscopy (TEM) micrographs were obtained with the Themis-Z 3.1 instrument (TFS, Waltham, MA, USA) equipped with a X-FEG-monochromator and a CS/S double corrector, an accelerating voltage of 200 kV and with a JEM-2200FS transmission electron microscope (JEOL Ltd., Tokyo, Japan, acceleration voltage 200 kV, lattice resolution ~1 Å) equipped with a Cs-corrector. Elemental analysis was performed with a Super-X EDS detector (energy resolution of approximately 120 eV) in the HAADF-STEM mode. The samples for the TEM study were prepared by ultrasonic dispersing in ethanol and subsequent deposition of the suspension upon a “holey” carbon film supported on a copper grid.

Temperature-programmed reduction by H_2_ (TPR-H_2_) was carried out in a flow installation using a feed containing 10 vol.% H_2_ in Ar at a flow rate of 40 mL/min and a temperature ramp of 10 °C min^−1^ from 25 °C to 900 °C. Before reduction, samples were pretreated in O_2_ at 500 °C for 0.5 h. Product water was separated by freezing at −80 °C. The H_2_ concentration was determined by a thermal conductivity detector.

A Shimadzu IRTracer-100 spectrometer (Shimadzu, Kyoto, Japan) was used for recording FTIR spectra. The spectra of adsorbed CO at −196 °C and at room temperature were obtained in the 400–6000 cm^−1^ range, accumulating 200 scans at 4 cm^−1^ resolution. The samples were pressed into pellets with a size of 1 × 2 cm^2^. A sample placed into the IR cell was heated in a vacuum to 600 °C and then calcined in a hydrogen atmosphere (100 Torr of H_2_) for 1 h at this temperature. After that, the sample was evacuated to a residual gas pressure not higher than 10^−4^ Torr and cooled to room temperature. CO was adsorbed at −196 °C and at a CO pressure from 0.1 to 10 Torr. After obtaining the spectra at liquid nitrogen temperature, the sample was heated to room temperature and the corresponding spectra were recorded. The obtained spectra were normalized to the optical thickness of the pellets. To obtain differential spectra, the spectrum before CO adsorption was subtracted from the spectrum after adsorption. The analysis of IR spectra was carried out by decomposition of the corresponding IR bands into individual Gaussian components. The amount of surface sites of different types was estimated using the integral absorption coefficients from the integrated intensities of the characteristic absorption bands [79]. The error in measuring the sites amount was 20%.

The oxygen mobility and surface reactivity of the samples were investigated by the temperature-programmed isotope exchange (TPIE) of oxygen with C^18^O_2_. The experiments were carried out with the sample weight 50 mg in a flow quartz tube reactor with an inner diameter of 3 mm. The samples were preliminarily pretreated in He + 1% O_2_ flow (flow rate 25 mL/min) at 700 °C for 30 min and cooled down to room temperature in He + 1% CO_2_ flow (flow rate 25 mL/min). After the steady state was reached, the feed gas mixture was switched to a mixture of the same composition but containing a ^18^O label. The temperature was raised from 50 °C to 700 °C with a ramp of 5 °C/min. The effluent gases were analyzed by the UGA 200 mass spectrometer (Stanford Research Systems, Sunnyvale, CA, USA). The analysis of the ^18^O atomic fraction (*α*) and C^16^O^18^O molecular fraction (*f*_16–18_) responses was made for calculating the values of the heteroexchange rate (*R*), oxygen tracer diffusion coefficient (D*) and their effective activation energies (*E_a,R_*, *E_a,D_*) using a mathematical model described earlier [71,80,81].

### 3.3. Catalytic Studies in Methane Dry Reforming

The catalytic studies in methane dry reforming was performed at atmospheric pressure in a tubular quartz plug flow reactor with a reaction mixture of 15% CH_4_ + 15% CO_2_ in N_2_ at a temperature of 550–700 °C and a contact time of 10 ms. The catalyst (fraction 0.5–0.25 mm) was diluted by the quartz fraction of the same size in a 1:1 ratio. The temperature was changed stepwise from 700 to 550 °C by 50 °C, and the duration of each step was 30 min. The reduction pretreatment of catalysts was made in a stream of 5% H_2_ in He at 700 °C for 1 h. The analysis of reagent and product concentrations was made by a gas analyzer with IR sensors for CO, CO_2_ and CH_4_, and an electrochemical sensor for H_2_ (Boner LLC, Novosibirsk, Russia).

The stability tests in DRM over 8 h were carried out at 700 °C using the same reaction mixture. The temperature-programmed oxidation (TPO) experiments were carried out to estimate the amount of carbon formed during stability tests. The oxidizing mixture contained O_2_ (17% vol.)/N_2_. The temperature was increased from 25 to 850 °C, at a 10 °C/min heating rate. Effluent gas concentrations were measured by the same gas analyzer (Boner LLC, Novosibirsk, Russia).

## 4. Conclusions

A series of 5%Ni/Ce_1-x_Ti_x_O_2_ catalysts was prepared with nickel impregnation of mixed Ce–Ti oxides obtained via synthesis in supercritical isopropanol. All oxides have a cubic fluorite phase structure. Ti is incorporated into the fluorite structure. Small amounts of impurities of TiO_2_ or mixed Ce–Ti oxides appear with Ti introduction. Supported Ni is presented as the NiO or NiTiO_3_ perovskite phase.

Ti introduction increases total samples reducibility and results in stronger interaction of supported Ni with the oxide support. The fraction of rapidly replaced oxygen and the average tracer diffusion coefficient also increase. The number of metallic nickel sites estimated using FTIRS of adsorbed CO decreased with increasing Ti content.

All catalysts except Ni-CeTi0.45 demonstrate close activity in tests of dry reforming of methane. The lower activity of Ni-CeTi0.45 can be connected to Ni decoration with species of the titanium-containing oxide support or with oxide support rearrangement. The incorporation of Ti prevents detachment of Ni particles from the surface and their sintering during dry reforming of methane.

## Figures and Tables

**Table 1:** Textural and structural characteristics of mixed oxides and catalysts, calcined at 700 °C.

№	Abbreviation	Composition	S_BET_ *, m^2^/g	V_pore_ *, cm^3^/g	a, Å	d, nm
**Supports**						
1	CeO_2_	CeO_2_	35	0.085	5.411(2)	29
2	CeTi0.15	Ce_0.85_Ti_0.15_O_2_	26	0.157	5.400(1)	12.6(5)
3	CeTi0.25	Ce_0.75_Ti_0.25_O_2_	25	0.179	5.396(1)	12.2(4)
4	CeTi0.35	Ce_0.65_Ti_0.35_O_2_	18	0.146	5.394(1)	11.9(1)
5	CeTi0.45	Ce_0.55_Ti_0.45_O_2_	17	0.174	5.388(2)	10.8(2)
**Catalysts**						
6	Ni-CeO_2_	5%Ni/CeO_2_	25	0.052	5.410(1)	29
7	Ni-CeTi0.15	5%Ni/Ce_0.85_Ti_0.15_O_2_	18	0.164	5.402(1)	14.5(2)
8	Ni-CeTi0.25	5%Ni/Ce_0.75_Ti_0.25_O_2_	17	0.184	5.398(1)	13.5(1)
9	Ni-CeTi0.35	5%Ni/Ce_0.65_Ti_0.35_O_2_	13	0.163	5.394(2)	13.0(4)
10	Ni-CeTi0.45	5%Ni/Ce_0.55_Ti_0.45_O_2_	12	0.181	5.391(2)	12.1(5)

*—relative error 10%. a—lattice parameter of the fluorite phase estimated from XRD data. d—crystallite size of the fluorite phase estimated from XRD data.

**Table 2:** Amount of various types of surface carbonyl complexes with Ni^0^ estimated from FTIR spectra of adsorbed CO at −196 °C.

Sample	νCO, cm^−1^	Sites Amount, μmol/g	Bridging CO Amount, μmol/g	On-Top CO Amount, μmol/g	Total Amount, μmol/g
Ni-CeO_2_	1922	9	9	13	22
	2004	1			
	2082	12			
Ni-CeTi0.15	1895	7	10	16	26
	1930	3			
	2090	16			
Ni-CeTi0.25	1880	4	6	9	15
	1930	2			
	2048	2			
	2093	7			
Ni-CeTi0.35	1875	2	5	11	16
	1932	3			
	2095	11			
Ni-CeTi0.45	1920	2	2	4	6
	2095	4			

**Table 3:** H_2_ consumption during TPR for mixed oxides and catalysts.

№	Sample	H_2_ Consumption, mmol H_2_/g_cat_		Ni Reducibility, %
		Oxide	Ni/Oxide	
1	CeO_2_	0.82	1.5	85
2	CeTi0.15	1.28	1.96	88
3	CeTi0.25	1.5	2.12	82
4	CeTi0.35	1.68	2.28	80
5	CeTi0.45	1.77	2.38	82

**Table 4:** Oxygen tracer diffusion coefficient values (D*) at 600 °C and fractions with respect to overall oxygen (*θ*) according to the TPIE with C^18^O_2_ data modeling. Effective activation energy of oxygen diffusion (*E_a,D_*) = 150 kJ/mol.

Sample	D*_fast_|_600 °C_ [cm^2^/s]	*θ_fast_* [%]	D*_slow_|_600 °C_ [cm^2^/s]	*θ_slow_* [%]	D*_average_|_600 °C_ [cm^2^/s]
**Supports**					
CeO_2_	^-^	-	2.2 × 10^−13^	100	2.2 × 10^−13^
CeTi0.15	≈3 × 10^−12^	10	2.2 × 10^−13^	90	5 × 10^−13^
CeTi0.25		15		85	6.4 × 10^−13^
CeTi0.35		23		77	8.6 × 10^−13^
CeTi0.45		30		70	11 × 10^−13^
**Catalysts**					
Ni-CeO_2_	-	-	6.6 × 10^−13^	100	6.6 × 10^−13^
Ni-CeTi0.15	≈3 × 10^−12^	3	4.1 × 10^−13^	97	4.9 × 10^−13^
Ni-CeTi0.25		3	3.3 × 10^−13^	97	4.1 × 10^−13^
Ni-CeTi0.35		3		97	4.1 × 10^−13^
Ni-CeTi0.45		5	2.2 × 10^−13^	95	3.6 × 10^−13^

**Table 5:** Comparison of the amount of carbon formed during stability tests in the DRM reaction with literature data.

Sample	mg_C_/g_cat_	Reaction Conditions	Reference
Ni-CeO_2_	321	15% CH_4_ + 15% CO_2_ + N_2_, 700 °C, 8 h	This work
Ni-CeTi0.45	66.1		
5%Ni/	1314 (25 h)	15% CH_4_ + 15% CO_2_ + N_2_, 700 °C, 25 and 3 h	[71] *
Ce0.75Ti0.05Nb0.05Zr0.15O2	998 (3 h)		
5% Ni/CeO_2_	241	40% CH_4_ + 40% CO_2_ + N_2_, 800 °C, 50 h	[74] *
5% Ni/Ce_0.85_La_0.15_O_2_	20.4		
5% Ni/CeO_2_	80.4	20% CH_4_ + 20% CO_2_ + He, 750 °C, 12 h	[26]
5% Ni/Ce_0.8_Ti_0.2_O_2_	0.3		
5% Ni/Ce_0.5_Ti_0.5_O_2_	66.4		
5% Ni/CeO_2_	8.9	50%CH_4_ + 50%CO_2_, 700 °C, 24 h	[29]
5% Ni/75%CeO_2_-25%ZrO_2_	83		
8% Ni/ZrO_2_	370	55% CH_4_ + 35% CO_2_ + Ar, 750 °C, 10 h	[76] *
8% Ni/8.1% La_2_O_3_-ZrO_2_	661		
8% Ni/18.8% CeO_2_-ZrO_2_	695		
La-NiMgAlO	690 (750 °C)	CH_4_:CO_2_ = 1:1, 650, 700 and 750 °C, 300 h	[77] *
	540 (700 °C)		
	420 (650 °C)		
5% Ni/SiO_2_	1857	50% CH_4_ + 50% CO_2_, 800 °C, 10 h	[75] *
5% Ni/CeO_2_	667		
5% Ni/30%CeO_2_–SiO_2_	220		

* mg_C_/g_cat_ was recalculated by the authors.

## Data Availability

Not applicable.

## References

[1] Usman M., Daud W.M.A.W., Abbas H.F. (2015). Dry reforming of methane: Influence of process parameters—A review. Renew. Sustain. Energy Rev..

[2] Sharifianjazi F., Esmaeilkhanian A., Bazli L., Eskandarinezhad S., Khaksar S., Shafiee P., Yusuf M., Abdullah B., Salahshour P., Sadeghi F. (2022). A review on recent advances in dry reforming of methane over Ni- and Co-based nanocatalysts. Int. J. Hydrogen Energy.

[3] Zhang G., Liu J., Xu Y., Sun Y. (2018). A review of CH_4_-CO_2_ reforming to synthesis gas over Ni-based catalysts in recent years (2010–2017). Int. J. Hydrogen Energy.

[4] Pakhare D., Spivey J. (2014). A review of dry (CO_2_) reforming of methane over noble metal catalysts. Chem. Soc. Rev..

[5] Bradford M.C.J., Vannice M.A. (1999). CO_2_ reforming of CH_4_. Catal. Rev..

[6] Tsyganok A.I., Inaba M., Tsunoda T., Hamakawa S., Suzuki K., Hayakawa T. (2003). Dry reforming of methane over supported noble metals: A novel approach to preparing catalysts. Catal. Commun..

[7] Hu Y.H., Ruckenstein E. (2004). Catalytic Conversion of Methane to Synthesis Gas by Partial Oxidation and CO_2_ Reforming. Adv. Catal..

[8] Marinho A.L.A., Toniolo F.S., Noronha F.B., Epron F., Duprez D., Bion N. (2021). Highly active and stable Ni dispersed on mesoporous CeO_2_-Al_2_O_3_ catalysts for production of syngas by dry reforming of methane. Appl. Catal. B.

[9] Radlik M., Adamowska-Teyssier M., Krzton A., Kozieł K., Krajewski W., Turek W., Da Costa P. (2015). Dry reforming of methane over Ni/Ce_0.62_Zr_0.38_O_2_ catalysts: Effect of Ni loading on the catalytic activity and on H_2_/CO production. Comptes Rendus Chim..

[10] Damaskinos C.M., Zavašnik J., Djinovi’c P., Efstathiou A.M. (2021). Dry reforming of methane over Ni/Ce_0.8_Ti_0.2_O_2_-δ: The effect of Ni particle size on the carbon pathways studied by transient and isotopic techniques. Appl. Catal. B.

[11] Rostrup-Nielsen J.R. (1991). Promotion by poisoning. Stud. Surf. Sci. Catal..

[12] Zhang Q., Akri M., Yang Y., Qiao B. (2023). Atomically dispersed metals as potential coke-resistant catalysts for dry reforming of methane. Cell Rep. Phys. Sci..

[13] Vasiliades M.A., Damaskinos C.M., Djinović P., Pintar A., Efstathiou A.M. (2021). Dry reforming of CH_4_ over NiCo/Ce_0.75_Zr_0.25_O_2_-δ: The effect of Co on the site activity and carbon pathways studied by transient techniques. Catal. Commun..

[14] Torimoto M., Sekine Y. (2022). Effects of alloying for steam or dry reforming of methane: A review of recent studies. Catal. Sci. Technol..

[15] Vasiliades M.A., Djinovi P., Davlyatova L.F., Pintar A., Efstathiou A.M. (2018). Origin and reactivity of active and inactive carbon formed during DRM over Ni/Ce_0.38_Zr_0.62_O_2_-δ studied by transient isotopic techniques. Catal. Today.

[16] Aramouni N.A.K., Touma J.G., Tarboush B.A., Zeaiter J., Ahmad M.N. (2018). Catalyst design for dry reforming of methane: Analysis review. Renew. Sust. Energy Rev..

[17] Cai X., Hu Y.H. (2019). Advances in catalytic conversion of methane and carbon dioxide to highly valuable products. Energy Sci. Eng..

[18] Zhang R., Xia G., Li M., Wu Y., Nie H., Li D. (2015). Effect of support on the performance of Ni-based catalyst in methane dry reforming. J. Fuel Chem. Technol..

[19] Rogers J.L., Mangarella M.C., D’Amico A.D., Gallagher J.R., Dutzer M.R., Stavitski E., Miller J.T., Sievers C. (2016). Differences in the Nature of Active Sites for Methane Dry Reforming and Methane Steam Reforming over Nickel Aluminate Catalysts. ACS Catal..

[20] Chaudhary P.K., Deo G. (2023). Process and catalyst improvements for the dry reforming of methane. Chem. Eng. Sci..

[21] Zhu J.Q., Peng X.X., Yao L., Deng X.J., Dong H.Y., Tong D.M. (2013). Synthesis gas production from CO_2_ reforming of methane over Ni-Ce/SiO_2_ catalyst: The effect of calcination ambience. Int. J. Hydrogen Energy.

[22] Salaev M.A., Liotta L.F., Vodyankina O.V. (2022). Lanthanoid-containing Ni-based catalysts for dry reforming of methane: A review. Int. J. Hydrogen Energy.

[23] Yuan B., Zhu T., Han Y., Zhang X., Wang M., Li C. (2023). Deactivation Mechanism and Anti-Deactivation Measures of Metal Catalyst in the Dry Reforming of Methane: A Review. Atmosphere.

[24] Ren Y., Ma Y.-Y., Mo W.-L., Guo J., Liu Q., Fan X., Zhang S.-P. (2023). Research Progress of Carbon Deposition on Ni-Based Catalyst for CO_2_-CH_4_ Reforming. Catalysts.

[25] Sadovskaya E.M., Ivanova Y.A., Pinaeva L.G., Grasso G., Kuznetsova T.G., van Veen A., Sadykov V.A., Mirodatos C. (2007). Kinetics of Oxygen Exchange over CeO_2_-ZrO_2_ Fluorite-Based Catalysts. J. Phys. Chem. A.

[26] Damaskinos C.M., Vasiliades M.A., Efstathiou A.M. (2019). The effect of Ti^4+^ dopant in the 5 wt% Ni/Ce_1-x_Ti_x_O_2-δ_ catalyst on the carbon pathways of dry reforming of methane studied by various transient and iso-topic techniques. Appl. Catal. A.

[27] Leitenburg C., Trovarelli A., Kaspar J. (1997). A Temperature-Programmed and Transient Kinetic Study of CO_2_ Activation and Methanation over CeO_2_ Supported Noble Metals. J. Catal..

[28] Luo M., Chen J., Chen L., Lu J., Feng Z., Li C. (2001). Structure and Redox Properties of Ce_x_Ti_1-x_O_2_ Solid Solution. Chem. Mater..

[29] Kambolis A., Matralis H., Trovarelli A., Papadopoulou C. (2010). Ni/CeO_2_-ZrO_2_ catalysts for the dry reforming of methane. Appl. Catal. A.

[30] Zhang F., Liu Z., Chen X., Rui N., Betancourt L.E., Lin L., Xu W., Sun C., Abeykoon A.M.M., Rodriguez J.A. (2020). Effects of Zr Doping into Ceria for the Dry Reforming of Methane over Ni/CeZrO_2_ Catalysts: In Situ Studies with XRD, XAFS, and APXPS. ACS Catal..

[31] Makri M.M., Vasiliades M.A., Petallidou K.C., Efstathiou A.M. (2016). Effect of support composition on the origin and reactivity of carbon formed during dry reforming of methane over 5wt%Ni/Ce1−xMxO_2_−I (M = Zr^4+^, Pr^3+^) catalysts. Catal. Today.

[32] Menegazzo F., Pizzolitto C., Ghedini E., Michele A.D., Cruciani G., Signoretto M. (2018). Development of La Doped Ni/CeO_2_ for CH_4_/CO_2_ Reforming. J. Carbon Res..

[33] Nagaoka K., Okamura M., Akia K. (2001). Titania supported ruthenium as a coking resistant catalyst for high pressure dry reforming of methane. Catal. Commun..

[34] Bradford M.C.J., Vannice M.A. (1999). The role of metal-support interactions in CO_2_ reforming of CH_4_. Catal. Today.

[35] Dutta G., Waghmare U.V., Baidya T., Hegde M.S., Priolkar K.R., Sarode P.R. (2006). Origin of Enhanced Reducibility/Oxygen Storage Capacity of Ce1-xTixO_2_ Compared to CeO_2_ or TiO_2_. Chem. Mater..

[36] Kim S.S., Lee S.M., Won J.M., Yang H.J., Hong S.C. (2015). Effect of Ce/Ti ratio on the catalytic activity and stability of Ni/CeO_2_–TiO_2_ catalyst for dry reforming of methane. Chem. Eng. J..

[37] Zhang Y., Li Z., Wen X., Liu Y. (2006). Partial oxidation of methane over Ni/Ce-Ti-O catalysts. Chem. Eng. J..

[38] Ye J.L., Wang Y.Q., Liu Y., Wang H. (2008). Steam reforming of ethanol over Ni/CexTi1-xO_2_ catalysts. Int. J. Hydrogen Energy.

[39] Simonov M., Bespalko Y., Smal E., Valeev K., Fedorova V., Krieger T., Sadykov V. (2020). Nickel-Containing Ceria-Zirconia Doped with Ti and Nb. Effect of Support Composition and Preparation Method on Catalytic Activity in Methane Dry Reforming. Nanomaterials.

[40] Arapova M., Smal E., Bespalko Y., Fedorova V., Valeev K., Cherepanova S., Ischenko A., Sadykov V., Simonov M. (2021). Ethanol dry reforming over Ni supported on modified ceria-zirconia catalysts: The effect of Ti and Nb dopants. Int. J. Hydrogen Energy.

[41] Aymonier C., Loppinet-Serani A., Reveron H., Garrabos Y., Cansell F. (2006). Review of supercritical fluids in inorganic materials science. J. Supercrit. Fluids.

[42] Leofanti G., Padovan M., Tozzola G., Venturelli B. (1998). Surface area and pore texture of catalysts. Catal. Today.

[43] Shannon R.D. (1976). Revised effective ionic radii and systematic studies of interatomic distances in halides and chalcogenides. Acta Crystallogr. Sect. A Cryst. Phys. Diffr. Theor. Gen. Crystallogr..

[44] Lu F., Jiang B., Wang J., Huang Z., Liao Z., Yang Y., Zheng J. (2017). Promotional effect of Ti doping on the ketonization of acetic acid over a CeO_2_ catalyst. RSC Adv..

[45] McBride J.R., Hass K.C., Poindexter B.D., Weber W.H. (1994). Raman and X-ray studies of Ce_1-x_RE_x_O_2-y_, where RE = La, Pr, Nd, Eu, Gd, and Tb. J. Appl. Phys..

[46] Kosacki I., Suzuki T., Anderson H.U., Colomban P. (2002). Raman scattering and lattice defects in nano-crystalline CeO_2_ thin films. Solid State Ion..

[47] Zhang Z., Han D., Wei S., Zhang Y. (2010). Determination of active site densities and mechanisms for soot combustion with O_2_ on Fe-doped CeO_2_ mixed oxides. J. Catal..

[48] Hu C., Zhu Q., Jiang Z. (2009). Nanosized CuO–ZrxCe1−xOy aerogel catalysts prepared by ethanol supercritical drying for catalytic deep oxidation of benzene. Powder Technol..

[49] Li Z., Li L., Yuan Q., Feng W., Xu J., Sun L., Song W., Yan C. (2008). Sustainable and Facile Route to Nearly Monodisperse Spherical Aggregates of CeO_2_ Nanocrystals with Ionic Liquids and Their Catalytic Activities for CO Oxidation. J. Phys. Chem. C.

[50] Zhang W.F., He Y.L., Zhang M.S., Yin Z., Chen Q. (2000). Raman scattering study on anatase TiO_2_ nanocrystals. J. Phys. D Appl. Phys..

[51] Zhang M.-S., Yin Z., Chen Q., Xijun W., Xiaoli J. (1995). Raman scattering by nanophase titanium dioxide. Ferroelectrics.

[52] Wu M., Zhang W., Du Z., Huang Y. (1999). Structural transformation in nanophase titanium dioxide. Mod. Phys. Lett. B.

[53] Romero-Nunez A., Dıaz G. (2015). High oxygen storage capacity and enhanced catalytic performance of NiO/Ni_x_Ce_1-x_O_2_ nanorods: Synergy between Ni-doping and 1D morphology. RSC Adv..

[54] Busca G., Ramis G., Amores J.M.G., Escribano V.S., Piaggio P. (1994). FT Raman and FTIR studies of titanias and metatitanate powders. J. Chem. Soc. Faraday Trans..

[55] Baraton M.I., Busca G., Prieto M.C., Ricchiardi G., Escribano V.S. (1994). On the Vibrational Spectra and Structure of FeCrO_3_ and of the Ilmenite-Type Compounds CoTiO_3_ and NiTiO_3_. J. Solid State Chem..

[56] Bespalko Y., Smal E., Simonov M., Valeev K., Fedorova V., Krieger T., Cherepanova S., Ishchenko A., Rogov V., Sadykov V. (2020). Novel Ni/Ce(Ti)ZrO_2_ Catalysts for Methane Dry Reforming Pre-pared in Supercritical Alcohol Media. Energies.

[57] Sadykov V.A., Kuznetsova T.G., Alikina G.M., Frolova Y.V., Lukashevich A.I., Potapova Y.V., Muzykantov V.S., Rogov V.A., Kriventsov V.V., Kochubei D.I. (2004). Ceria-based fluorite-like oxide solid solutions as catalysts of methane selective oxidation into syngas by the lattice oxygen: Synthesis, characterization and performance. Catal. Today.

[58] Kuznetsova T.G., Sadykov V.A., Moroz E.M., Trukhan S.N., Paukshtis E.A., Kolomiichuk V.N., Burgina E.B., Zaikovskii V.I., Fedotov M.A., Lunin V.V. (2002). Preparation of Ce-Zr-O composites by a polymerized complex method. Stud. Surf. Sci. Catal..

[59] Romero-Galarza A., Dahlberg K.A., Chen X., Schwank J.W. (2014). Crystalline structure refinements and properties of Ni/TiO_2_ and Ni/TiO2-Ce catalysts and application to catalytic reaction of “CO + NO”. Appl. Catal. A.

[60] Busca G., Saussey H., Saur O., Lavalley J.-C., Lorenzelli V. (1985). FT-IR characterization of the surface acidity of different titanium dioxide anatase preparations. Appl. Catal..

[61] Hadjiivanov K.I., Klissurski D.G. (1996). Surface Chemistry of Titania (Anatase) and Titania-supported Catalysts. Chem. Soc. Rev..

[62] Manoilova O.V., Dakka J., Sheldon R.A., Tsyganenko A.A. (1995). Infrared study of Ti-containing zeolites using CO as a probe molecule. Stud. Surf. Sci. Catal..

[63] Sadykov V.A., Kuznetsova T.G., Alikina G.M., Frolova Y.V., Lukashevich A.I., Muzykantov V.S., Rogov V.A., Batuev L.C., Kriventsov V.V., Kochubei D.I., McReynolds D.K. (2007). Ceria-based fluorite-like oxide solid solutions promoted by precious metals as catalysts of methane transformation into syngas. New Topics in Catalysis Research.

[64] Hadjiivanov K.I., Vayssilov G.N. (2002). Characterization of oxide surfaces and zeolites by carbon monoxide as an IR probe molecule. Adv. Catal..

[65] Bernal S., Calvino J.J., Cauqui M.A., Gatica J.M., López Cartes C., Pérez Omil J.A., Pintado J.M. (2003). Some contributions of electron microscopy to the characterization of the strong metal–support interaction effect. Catal. Today.

[66] Bernal S., Botana F.J., Calvino J.J., Lopez C., Perez-Omil J.A., Rodriguez-Izquierdo J.M. (1996). High-resolution electron microscopy investigation of metal-support interactions in Rh/TiO2. J. Chem. Soc. Faraday Trans..

[67] Zhu H., Qin Z., Shan W., Shen W., Wang J. (2004). Pd/CeO_2_–TiO_2_ catalyst for CO oxidation at low temperature: A TPR study with H_2_ and CO as reducing agents. J. Catal..

[68] Li J., Li L., Wu F., Zhang L., Liu X. (2013). Dispersion–precipitation synthesis of nanorod Mn_3_O_4_ with high reducibility and the catalytic complete oxidation of air pollutants. Catal. Commun..

[69] Shan W., Luo M., Ying P., Shen W., Li C. (2003). Reduction property and catalytic activity of Ce_1-X_Ni_X_O_2_ mixed oxide catalysts for CH_4_ oxidation. Appl. Catal. A.

[70] Montoya J.A., Romero-Pascual E., Gimon C., Del Angel P., Monzón A. (2000). Methane reforming with CO_2_ over Ni/ZrO_2_-CeO_2_ catalysts prepared by sol-gel. Catal. Today.

[71] Smal E., Bespalko Y., Arapova M., Fedorova V., Valeev K., Eremeev N., Sadovskaya E., Krieger T., Glazneva T., Sadykov V. (2022). Car-bon Formation during Methane Dry Reforming over Ni-Containing Ceria-Zirconia Catalysts. Nanomaterials.

[72] Frusteri F., Freni S., Chiodo V., Donato S., Bonura G., Cavallaro S. (2006). Steam and auto-thermal reforming of bio-ethanol over MgO and CeO_2_ Ni-supported catalysts. Int. J. Hydrogen Energy.

[73] Li M., van Veen A.C. (2018). Tuning the catalytic performance of Ni-catalysed dry reforming of methane and carbon deposition via Ni-CeO_2_-x interaction. Appl. Catal. B.

[74] Luisetto I., Tuti S., Romano C., Boaro M., Di Bartolomeo E., Kesavan J.K., Kumar S.S., Selvakumar K. (2019). Dry reforming of methane over Ni supported on doped CeO_2_: New insight on the role of dopants for CO_2_ activation. J. CO_2_ Util..

[75] Taufiq-Yap Y.H., Sudarno, Rashid U., Zainal Z. (2013). CeO_2_–SiO_2_ supported nickel catalysts for dry re-forming of methane toward syngas production. Appl. Catal. A.

[76] Charisiou N.D., Siakavelas G., Tzounis L., Sebastian V., Monzon A., Baker M.A., Hinder S.J., Polychronopoulou K., Yentekakis I.V., Goula M.A. (2018). An in depth investigation of deactivation through carbon formation during the biogas dry reforming reaction for Ni supported on modified with CeO_2_ and La_2_O_3_ zirconia catalysts. Int. J. Hydrogen Energy.

[77] Serrano-Lotina A., Daza L. (2014). Long-term stability test of Ni-based catalyst in carbon dioxide reforming of methane. Appl. Catal. A.

[78] Fedorova V., Bespalko Y., Arapova M., Smal E., Valeev K., Prosvirin I., Sadykov V., Parkhomenko K., Roger A.-C., Simonov M. (2023). Ethanol Dry Reforming over Bimetallic Ni-Containing Catalysts Based on Ceria-Zirconia for Hydrogen Production. ChemCatChem.

[79] Paukshtis E.A., Yurchenko E.N. (1983). Study of the Acid–Base Properties of Heterogeneous Catalysts by Infrared Spectroscopy. Russ. Chem. Rev..

[80] Fedorova V., Simonov M., Valeev K., Bespalko Y., Smal E., Eremeev N., Sadovskaya E., Krieg-er T., Ishchenko A., Sadykov V. (2021). Kinetic regularities of methane dry reforming reaction on nickel-containing modified ceria–zirconia. Energies.

[81] Sadykov V.A., Sadovskaya E.M., Uvarov N.F. (2015). Methods of isotopic relaxations for estimation of oxygen diffusion coefficients in solid electrolytes and materials with mixed ionic-electronic conductivity. Russ. J. Electrochem..

